# Relationship between Helicobacter pylori infection (HP) and bone mineral density (BMD) in elderly people

**Published:** 2015

**Authors:** Mahdiye Fotouk-kiai, Seyed Reza Hoseini, Neda Meftah, Reza Ghadimi, Ali Bijani, Hajighorban Noreddini, Hamidreza Nematollahi, Javad Shokri-Shirvani

**Affiliations:** 1 Department of Internal Medicine, Babol University of Medical Sciences, Babol, Iran.; 2 Social Determinates of Health Research Center, Health Research Institute, Ba Babol University of Medical Sciences Babol, Iran.; 3 Department of Radiology, Babol University of Medical Sciences, Babol, Iran.

**Keywords:** Helicobacter pylori, Osteoprosis, Bone mineral density, Elderly patients

## Abstract

**Background:**

Low bone mass is a frequent complication of chronic inflammatory disease. The pathogenesis of osteoporosis in chronic inflammatory disease may be secondary to releases of cytokines such as TNF- and IL6. Chronic gastritis due to *helicobacter pylori* (HP) infection may lead to decreased bone mineral density (BMD) and predispose patients to osteoporosis. The objective of this study was to determine the BMD status in HP positive patients with gastritis versus HP negative cases.

**Methods:**

In this cross-sectional study, we enrolled 967 participants aged 60 years old and more from Amirkola Health Study Ageing Project. Seven-hundred and fifty eight HP positive and 209 HP negative patients were analyzed. BMD was measured by dual-energy x-ray absorptiometry (DXA) method in the spine and femoral neck in all participants.

**Results:**

The mean age in HP+ and HP- negative patients was 68.3±6.8 and 69.3±7.4 years, respectively. BMD g/cm2 in the spine and femoral neck did not differ between the two groups (P=0.19 and 0.22 respectively). The prevalence of osteoporosis did not also differ across the two groups as well. There was no relationship between the level of antibodies against HP and BMD.

**Conclusion:**

According to the findings of this study, H. pylori infection is not associated with BMD changes in the elderly population.

Osteoporosis is defined as the reduction of bone mineral density, which is common in postmenopausal women. But it also occurs in men and women with the underlying causes or risk factors related to bone demineralization (1). Osteoporosis is the most common metabolic bone disease (2); and caused by bone density loss due to age-related changes during bone formation period as well as the internal and external factors aggravating the process. 

These changes may be added to the low peak bone mass (1, 3). Several underlying diseases, including genetic and the acquired ones can reduce bone mineral content and increase the risk of osteoporosis; among which are the endocrine disorders, organ transplantation, chronic inflammatory diseases, cancer and gastrointestinal diseases and taking some medications (1). Among the many factors that play a role in osteoporosis and bone loss, inflammation is the important one. Inflammation exerts a significant effect on bone mass. Several cytokines regulate osteoblasts and osteoclasts (4). Chronic inflammatory diseases decrease bone formation and increase bone resorption (5).

The relation of several gastrointestinal diseases such as malabsorption syndrome, gastrectomy, inflammatory bowel disease (IBD) and taking some medications such as proton pump inhibitors (PPI) with bone density loss have been shown (1, 2, 6). Chronic gastritis due to HP is chronic inflammatory disease which persists for a long time, gastritis may release several cytokines resulting in bone loss. In chronic gastritis eradication of HP infection can reduce inflammation significantly (7-11). In addition, HP infection may be associated with a number of endocrine disorders (10, 12-14).

Considering the high prevalence of HP infection in Mazandaran of Iran (78%) (15), so this study was designed to find any association between this infection and loss of bone density in this region. The objective of this study was to determine BMD in patients with helicbacter pylori infection versus HP negative participants.

## Methods

This research is part of a comprehensive Amirkola Health and Ageing Project (AHAP) which was done on 1616 participants about 60 years and older between 2011-2012 in Amirkola town (16). Based on the exclusion criteria, 649 patients were excluded. And finally, 967 patients were eligible for the study. Exclusion criteria included the following: history of chronic inflammatory diseases (RA, SLE, AS, IBD), history of cancer, history of antibiotic treatment affecting H. pylori during the past two months, history of diseases affecting bone metabolism (chronic liver disease - chronic kidney disease – hyperthyroidism), history of drugs like calcium - vitamin D - Fluoride - bisphosphonates - calcitonin - HRT in recent years, and a total vegetarian diet. For the inclusion criteria in the study, besides the completion of the questionnaire based on the absence of exclusion criteria, the blood samples were taken and the level of anti-HP IgG antibody was measured by ELISA method using Patton kits made in Iran. They were also referred to the department of radiology to investigate the density of bone and bone mineral density (BMD) of the lumbar spine (L1-L5) and femoral neck were measured by LEXXOS made in France Model 2007 and was shown with BMD (gr / cm2); and T-Score <-2.5 was considered as osteoporosis. Besides BMD, height and weight were measured and BMI was calculated as well. The study population, according to the level of antibodies against H.pylori, were divided into two HP+ (positive antibody against helicobacter pylori) (IgG antibody level> 20UR/ml) and control or HP – (negative antibody) (IgG antibody level <20UR/ml) groups. Then BMD was compared between HP^+^ and HP^-^ groups. T-score and Z-score in spine area and femoral neck and the prevalence of osteoporosis was calculated in each group and compared between the two groups after adjustment of some factors such as sex, age, BMI, smoking and alcohol consumption. Data were analyzed by SPSS Version 17 with t-test, chi-square, Pearson correlation and logistic regression models and p<0.05 was considered significant.

## Results

Within a year (2011-2012), after the removal of patients with exclusion criteria, 758 patients were selected as HP+ and 209 patients as a control or HP- group. The average age in HP + group was 68.3±6.8 and in HP- group was 69.3±7.4 years (p=0.069). In HP positive patients 59.9% were males but 57.9% in HP negative group were males (p=0.6). Mean BMI was 27.275±4.7 and 26.88±4.5 in HP+ and HP- patients respectively (p=0.28). 

Comparing the mean BMD of the lumbar spine and femoral neck in both groups, the difference was not significant. Also, a comparison of the mean t-score in femoral neck between the two groups did not show any significant difference. However, the mean T-score of the lumbar spine in the HP+ was significantly higher than HP- group unexpectedly (P=0.009) (table 1). 

**Table 1:** Comparison of bone density (BMD) at the femoral neck and lumbar spine in Helicobacter pylori positive (HP+) and negative (HP-) elderly participant of amirkola City

**PValue**	**HP** ^-^ **Mean±SD**	**HP** ^+^ **Mean±SD**	**Variable**
0.19	0.845±0.19	0.879±0.18	Lumbar vertebra BMD
0.16	-0.618±1.3	-0.370±1.3	Z-score
0.009	1.8474±1.1	-1.545±1.1	T-score
0.22	0.841±0.16	0.855±1.15	Femur BMD
0.29	-0.572±1.06	-0.490±0.98	Z-score
0.17	-1.488±1.2	-1.366±1.1	T-score

BMD: bone mineral density

**Figure 1:**
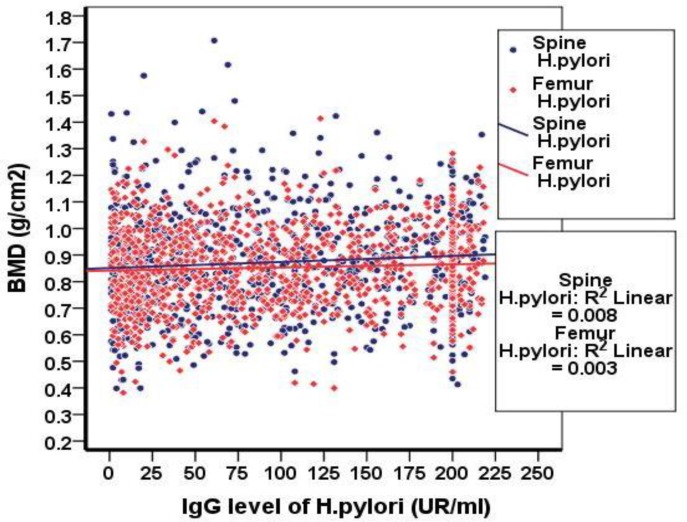
The relationship between HP antibody and bone density levels

But, according to the findings and considering the bone density and T-score, the prevalence of osteoporosis was not significantly different in the two groups and no relationship between the level of antibodies against HP and BMD was observed (figure 1). After adjusting the effective factors in osteoporosis (age, sex, smoking, alcohol consumption and BMI). In patients with positive HP infection 522 (68.9%) and in HP negative group 131 (62.7%) were not osteoporotic (CI95%: 0.55-1.045) OR=0.759) (figure 1).

**Figure 2:**
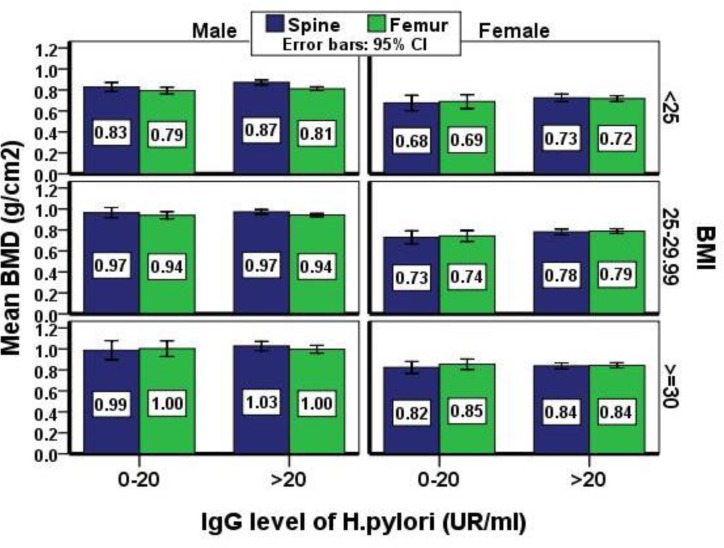
Comparison between the femor and lumbar spine BMD between the sexes, BMI groups and anti-helicobacter pylori antibody levels

## Discussion

In this study, the comparison of HP + and HP- groups showed that the femoral bone density was not significantly different between the two groups. Even the bone density in the lumbar spine was significantly more in group HP + than HP- unexpectedly, which was in contrast with the results of the Abravesh et al.’s study in 2011 in Babol (11). Also, the results of this study showed that the prevalence of osteoporosis was not significantly different among these two groups; so it can be concluded that the level of anti-HP antibodies does not cause a significant decrease in bone density. Bone density is the most important element in increasing stamina and withstanding the pressure on bones which is influenced by many factors, such as chronic inflammatory disease, cancer, gender, drugs, smoking and alcohol. In this study, a number of these factors (exclusion criteria) were excluded and the impact of factors such as gender, age and BMI was adjusted. Also, because there was no accurate information about the time of alcohol consumption and smoking rates, the impact of these factors was adjusted. However, despite adjusting the effect of these factors, the prevalence of osteoporosis was not significantly different between the two groups. So far, several chronic inflammatory diseases, including IBD and RA have been shown to be effective in reducing bone density and can increase or intensify the prevalence of osteoporosis. The main cause of a decrease in bone density in these conditions was the production of inflammatory cytokines that can cause bone loss by affecting bone metabolism and calcium absorption. Numerous studies have been conducted in this area and in all of them, the presence of inflammatory cytokines affecting bone density reduction (IL6, TNF α) was confirmed. 

Chronic gastritis caused by HP infection is a chronic inflammation which may involve the gastric mucosa and can cause the production of inflammatory cytokines by recalling inflammatory cells. Entering these cytokines into the blood and to various organs, raises the question of whether HP infection has also the ability to cause bone deformities or not? Some studies have not shown the increased levels of biochemical markers of bone metabolism in children with Helicobacter pylori infection; but they represent an increase in the prevalence of Cag A + Helicobacter pylori infection in men who have been diagnosed with osteoporosis. In our study, these biochemical markers were not measured. The best way to confirm the chronic infection of Helicobacter Pylori is to find the bacteria in the gastric mucosa. But based on the invasive nature of this procedure and the high sample size of this study, the method was used to measure the levels of anti-HP IgG antibodies which has the sensitivity> 80% and specificity> 90% in the diagnosis of infection and is easy and inexpensive and common (1). 

The results of our study are similar to the most other studies in this field. In previous studies, it was observed that infection with HP except in species which has CagA + protein was not a risk factor for osteoporosis in men. Some studies have illustrated that neither chronic atrophic gastritis nor HP infection was not the reliable risk factor for osteoporosis in postmenopausal women. Also, the bone density in patients with autoimmune gastritis and gastritis caused by HP was not significantly different from the control group. However, a study showed that a significant reduction of spine density in women under 50 years had no significant difference between chronic inflammation caused by Helicobacter pylori (which was proven by biopsy) and also there was no anti-Helicobacter pylori antibody with decreased bone density and osteoporosis was found and it was observed that these infections can lead to loss of bone density.

In this study, we found that Helicobacter pylori infection does not increase the osteoporotic chance and is not a reliable risk factor for osteoporosis but T-score in lumbar spine in HP^+ ^group was significantly higher than HP^- ^group. This observation should be explained by different distribution of lumbar spine osteoarthritis across the two comparison groups. 

The presence of lumbar spine osteoarthritis which falsely increases spine BMD (17). This study has several limitations regarding the study population who are elderly patients of AHAP project. A proportion of these patients have limited physical activity due to presence or complications of knee osteoarthritis (18, 19). In addition the number of parity and duration of menopause and presence of inflammatory diseases like rheumatoid arthritis which has not been reported by the patients may differently affect BMD across the two groups (20-22).

 Perhaps the result of our study is because of the level of antibodies, however, it was the immune response, but does not specify the degree of inflammation as well. But due to the high number of cases in our study, none of the previous studies in this area had-lack of relationship between helicobacter pylori infection and osteoporosis in the size of the sample can certainly prove that there is no relationship.

HP^- ^group may be affected with helicobacter pylori infection in the past years and were treated. This inflammation may effect on the decreasing bone density in this group and may cause no significant difference between bone density in two groups.

Also, because the HP antibody may have high level after treatment, confirmation of HP infection with biopsy and pathology seems to be more accurate method. But due to large number of our participants, it was time consuming and expensive.

## Notes

**Funding: **This project was financially supported by Babol University of Medical Sciences.
